# Circ_0001421 facilitates glycolysis and lung cancer development by regulating miR-4677-3p/CDCA3

**DOI:** 10.1186/s13000-020-01048-1

**Published:** 2020-10-28

**Authors:** Koudong Zhang, Hang Hu, Juan Xu, Limin Qiu, Haitao Chen, Xingzhi Jiang, Yongqian Jiang

**Affiliations:** 1 Department of Respiratory and Critical Medicine, The Fourth Affiliated Hospital of Nantong University; The First People’s Hospital of Yancheng, No.66 Renmin South Road, Yancheng, 224000 Jiangsu China; 2 Department of Cardiothoracic Surgery, The Fourth Affiliated Hospital of Nantong University; The First People’s Hospital of Yancheng, Yancheng, 224000 Jiangsu China; 3 Department of Pathology, The Fourth Affiliated Hospital of Nantong University; The First People’s Hospital of Yancheng, Yancheng, 224000 Jiangsu China

**Keywords:** Lung cancer, circ_0001421, miR-4677-3p, CDCA3

## Abstract

**Background:**

Lung cancer (LC) is a malignant tumor originating in the bronchial mucosa or gland of the lung. Circular RNAs (circRNAs) are proved to be key regulators of tumor progression. However, the regulatory effect of circ_0001421 on lung cancer tumorigenesis remains unclear.

**Methods:**

The expression levels of circ_0001421, microRNA-4677-3p (miR-4677-3p) and cell division cycle associated 3 (CDCA3) were detected by quantitative real-time polymerase chain reaction (qRT-PCR). Methyl thiazolyl tetrazolium (MTT), Transwell and Tumor formation assays were performed to explore the role of circ_0001421 in LC. Glucose consumption and lactate production were examined by a Glucose assay kit and a Lactic Acid assay kit. Western blot was utilized to examine the protein levels of Hexokinase 2 (HK2) and CDCA3. The interaction between miR-4677-3p and circ_0001421 or CDCA3 was confirmed by dual-luciferase reporter assay.

**Results:**

Circ_0001421 was increased in LC tissues and cells, and knockdown of circ_0001421 repressed cell proliferation, migration, invasion and glycolysis in vitro. Meanwhile, circ_0001421 knockdown inhibited LC tumor growth in vivo. Mechanistically, circ_0001421 could bind to miR-4677-3p, and CDCA3 was a target of miR-4677-3p. Rescue assays manifested that silencing miR-4677-3p or CDCA3 overexpression reversed circ_0001421 knockdown-mediated suppression on cell proliferation, migration, invasion and glycolysis in LC cells.

**Conclusion:**

Circ_0001421 promoted cell proliferation, migration, invasion and glycolysis in LC by regulating the miR-4677-3p/CDCA3 axis, which providing a new mechanism for LC tumor progression.

## Background

Lung cancer (LC) is a malignant tumor with the highest morbidity and mortality in worldwide [1, 2]. According to histopathological classification, LC included non-small cell lung cancer (NSCLC) and small cell lung cancer. The 5-year survival rate of LC patients is only 17.7%, mainly on account of the rapid tumor metastasis and local recurrence [3]. Besides, the 5-year survival rate of patients with early-stage LC is significantly higher than that of advanced LC patients [4]. Hence, it is necessary to dive deep into the underlying mechanism of lung cancer tumorigenesis and progression.

Circular RNA (circRNA) is a special form of non-coding RNA (ncRNA) that form a loop with jointed 3′ heads and 5′ tails [5]. CircRNAs have received attention due to their conservation and tissue specific expression in living beings [6]. The regulatory mechanism of circRNAs has been further investigated, and some circRNAs play regulatory roles by serving as sponges to adsorb microRNAs (miRNAs) [7]. A number of circRNAs were involved in cancer development. For example, circNRIP1 boosted cell growth, migration and invasion through sponging miR-149-5p in gastric cancer [8]. Silencing circDENND4C repressed breast cancer cell glycolysis and metastasis by sponging miR-200b/c under hypoxia [9]. The effects of circRNAs on LC have also been reported. CircFGFR1 could elevate cell proliferation and invasion by targeting miR-381-3p in NSCLC [10]. CircRNA SMARCA5 played an inhibition role in the progression of LC by acting as miR-19b-3p sponge [11]. Circular RNA, circ_0001421 (circ-SEC31A) was upregulated in NSCLC and its high expression predicts unfavorable prognoses [12]. However, the role of circ_0001421 in lung cancer tumorigenesis and progression was needed to be elucidated further.

MiRNAs are also ncRNA molecules with 18 ~ 25 nucleotides in length, and they play a biological role by targeting the downstream mRNAs [13]. As reported, miR-196b-5p promoted tumor development in NSCLC by targeting TSPAN12 and GATA6 [14]. MiR-212-5p enhanced tumor growth by regulating Id3 in lung adenocarcinoma cells [15]. MiR-4677-3p was found to be decreased in lung adenocarcinoma tissue samples, and lncRNA TTN-AS1 elevated cell migration and invasion by acting as miR-4677-3p sponge [16], whereas the interaction between miR-4677-3p and circ_0001421 was not studied in LC.

Cell division cycle associated protein-3 (CDCA3) modulates cell cycle processes, also called as trigger of mitosis entry 1, and it was identified to play a pro-carcinogenic role in gastric cancer [17], colorectal cancer [18] and acute myeloid leukemia [19]. However, the function of CDCA3 in LC has been little studied, and its effect is not fully understood.

Here, we investigated the expression of circ_0001421 in LC and its effects on cell proliferation, migration, invasion and glycolysis. Additionally, we appraised the interaction between circ_0001421 and miR-4677-3p, as well as the downstream genes. In conclusion, the aim of this study was to explore the role of circ_0001421 in LC progression.

## Materials and methods

### Clinical tissues and cell

The 48 paired lung cancer tissues and adjacent normal tissues were collected from lung cancer patients, who were diagnosed in The Fourth Affiliated Hospital of Nantong University; The First People’s Hospital of Yancheng from June 2016 to December 2018. The samples were immediately transferred to liquid nitrogen for further research. Written informed consents were obtained from these LC patients. And this study was reviewed and approved by The Fourth Affiliated Hospital of Nantong University; The First People’s Hospital of Yancheng. Table 1 presents the clinical characteristics.

**Table 1:** Correlation between hsa_circ_0001421 expression and clinicopathological characteristics in lung cancer

Clinical parameter	Hsa_circ_0001421 High (*n* = 24)	Hsa_circ_0001421 Low (n = 24)	*P*
Age (years)			
≤ 60	9	11	0.107
> 60	15	13	
Gender			
Male	14	12	0.116
Female	10	12	
Histological grade			
Low or undiffer	10	12	0.155
Middle or high	14	12	
TNM stages			
Iand II	15	16	0.018^*^
III and IV	9	8	
Size			
≤ 5 cm	8	7	0.015^*^
> 5 cm	16	17	
Invasion depth			
T1 and T2	14	19	0.033^*^
T3 and T4	25	12	
Lymphatic metastasis			
Yes	11	10	0.153
No	13	14	
Distant metastasis			
Yes	12	11	0.188
No	12	13	

Human LC cells (A549 and H1299) bought from American Type Culture Collection (ATCC, Manassas, VA, USA) and human normal bronchial epithelial cells (16HBE) bought from Procell (Wuhan, China) were maintained in Dulbecco’s Modified Eagle Medium (DMEM, Invitrogen, Carlsbad, CA, USA) containing 10% fetal bovine serum (FBS) at 37 °C with 5% CO_2_.

### Transfection

Small interfering RNA targeting circ_0001421 (si-circ_0001421, 5′-CCGGCTCTGGAGTTCTGATTGCATTCTCGAGTGCAATCAGAACTCCAGAGTTTTTTTG-3′) and negative control (si-NC, 5′-UUCUCCGAACGUGUCACUTT-3′), miR-4677-3p mimic (miR-4677-3p, 5′-UCAUCAAGAAACCAGAGUGUCU-3′), mimic NC (miR-NC, 5′-GAAAUGUACUUGAGCGUGGAGAC-3′), miR-4677-3p inhibitor (anti-miR-4677-3p, 5′-GUCUCCACGCUCAAGUACAUUUC-3′), inhibitor NC (anti-miR-NC, 5′-CUAAAACCGGCCGUACGGCGUU-3′), short-hairpin against circ_0001421 (sh-circ_0001421, 5′-CCGGGCTAAGATTCTCTGCTCCAATCTCGAGATTGGAGCAGAGAATCTTAGCTTTTTTG-3′) and control (sh-NC, 5′-UUCUCCGAACGUGUCACGUTT-3′) were acquired from GenePharma (Shanghai, China). Overexpression vector Vector-CDCA3 (CDCA3) and control (Vector) were acquired from GenePharma. Lipofectamine 3000 (Invitrogen) was employed to conduct cell transfection in this study.

### Quantitative real-time polymerase chain reaction (qRT-PCR)

The RNA was extracted from LC tissues and cells via TRIzol (Invitrogen), and then reversely transcribed to complementary DNA (cDNA) through the PrimeScript™ strand cDNA synthesis kit (Takara, Dalian, China). The reaction of qRT-PCR was carried out by a Power SYBRTM Green PCR Master Mix (Thermo Fisher Scientific, Waltham, MA, USA) on an ABI 7900 system (Thermo Fisher Scientific). U6 and glyceraldehyde-3-phosphate dehydrogenase (GAPDH) were used as the internal controls for miRNA and circRNA or mRNA, respectively. Primer sequences were listed as follows: circ_0001421, F: 5′-TCTCTGGAGTTCTGATTGCAGGTGG-3′ and R: 5′-TGCTAGGTAAATGGGGTGATTCTGG-3′; miR-4677-3p, F: 5′-CTGTGAGACCAAAGAACTACTCGC-3′ and R: 5′-CTCTACAGCTATATTGCCAGCCAC-3′; CDCA3, F: 5′-GGACCCTGAGACTCCCAGAT-3′ and R: 5′-GCCGCTTACCCTGTCGTAG-3′; GAPDH, F: 5′-CCATTTGCAGTGGCAAAG-3′ and R: 5′-CACCCCATTTGATGTTAGTG-3′; and U6, F: 5′-GCTTCGGCAGCACATATACTAAAAT-3′ and R: 5′-TACTGTGCGTTTAAGCACTTCGC-3′.

### Cell proliferation detection

The proliferation of A549 and H1299 cells was determined through the Methyl thiazolyl tetrazolium (MTT, Sigma-Aldrich, St. Louis, MO, USA) reagent. Cells were inoculated into the 96-well plates. 10 μL MTT was added into the cells. Then, 100 μL Dimethyl sulfoxide (DMSO, Sigma-Aldrich) was added to the cells. The absorbance at 490 nm was detected at 0 h, 24 h, 48 h and 72 h by a microplate reader (Bio-Rad, Hercules, CA, USA).

### Transwell assay

Transwell assay with the chamber was performed to assess cell migration (uncoated Matrigel) and invasion (coated Matrigel, Thermo Fisher Scientific). Transfected LC cells suspended in serum-free medium were seeded into the top chambers, and 600 μL medium with 10% FBS was added into the lower chamber. 24 h later, cells on the lower surface of chambers were fixed by methanol and then stained with 0.1% crystal violet for 20 min. Finally, a microscope was used to quantify the number of migrated or invaded cells.

### Glucose consumption and lactate production analysis

The transfected A549 and H1299 cells were harvested at 48 h post-transfection, and the cell culture medium were collected. Then, the concentration of glucose in cell culture medium was examined by a Glucose assay kit (Sigma-Aldrich, St. Louis, MO, USA), and lactate concentration was determined by a Lactic Acid assay kit (Seebio, Shanghai, China).

### Western blot

Proteins were extracted from cell and tissue lysates using RIPA buffer (Invitrogen), and then separated by sodium dodecyl sulfate polyacrylamide gel electrophoresis (SDS-PAGE). Subsequently, samples were transferred onto polyvinylidene fluoride (PVDF, Millipore, Billerica, MA, USA) membranes. 5% skim milk powder was used to block these membranes for 2 h. Then, the bands were incubated with the primary antibodies against Hexokinase 2 (HK2, 1:1000, Abcam, Cambridge, MA, USA), CDCA3 (1:1000, Abcam) or GAPDH (1:2000, Abcam) at 4 °C overnight. The next day, the membranes were mixed with the secondary antibodies (1:4000, Abcam) for 1 h. The immunoblots were detected with the enhanced chemiluminescence reagent (Millipore).

### Dual-luciferase reporter assay

To affirm the interaction between miR-4677-3p and circ_0001421 or CDCA3, the wild-type (WT) sequences of circ_0001421 contained miR-4677-3p binding sites or its mutant (MUT) were cloned into the pmirGLO vector (Promega, Madison, WI, USA) to form circ_0001421-WT or circ_0001421-MUT reporter vector. CDCA3–3’UTR-WT and CDCA3–3’UTR-MUT were constructed in the same way. These reporter vectors were co-transfected into cells with miR-4677-3p or miR-NC by using Lipofectamine 3000. 48 h later, the luciferase activity was determined by a Dual-Luciferase Reporter assay system (Promega).

### Xenograft tumors in nude mice

Twelve male nude BALB/c mice (20–22 g, 4 weeks) were purchased from Shanghai Laboratory Animal Center (SLAC, Shanghai, China). A549 cells transfected with sh-circ_0001421 or sh-NC were injected into the nude mice. Tumor volume was measured once a week. Five weeks later, all the mice were euthanatized with an overdose of ether, and tumor weight was measured. Western blot and qRT-PCR were performed to examine the expression of CDCA3 and circ_0001421 or miR-4677-3p, respectively.

### Statistical analysis

Data were displayed as the mean ± standard deviation (SD). The difference of two groups was evaluated by Wilcoxon test, and one-way analysis of variance (ANOVA) was applied for more than two groups. *P* < 0.05 was considered as statistically distinct. Each test was repeated at least three times.

## Result

### Circ_0001421 was overexpressed in LC tissues and cells

**Fig. 1:**
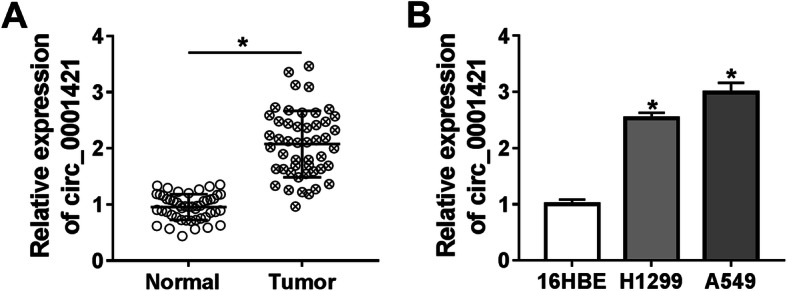
Circ_0001421 was overexpressed in LC tissues and cells. **a** Circ_0001421 expression in tumor and adjacent normal tissues was detected by qRT-PCR. **b** qRT-PCR was performed to examine circ_0001421 expression in LC cell lines (A549 and H1299) and normal human bronchial epithelial cells (16HBE). *P < 0.05

Firstly, circ_0001421 expression in tumor and normal tissues of 48 LC patients was measured by qRT-PCR. The results showed that circ_0001421 expression in tumor tissues was significantly higher than that in normal tissues (Fig. 1a). Additionally, circ_0001421 was overexpressed in LC cell lines, including A549 and H1299 cells, compared to 16HBE cells (Fig. 1b).

### Knockdown of circ_0001421 repressed LC cell proliferation, migration, invasion and glycolysis in vitro

**Fig. 2:**
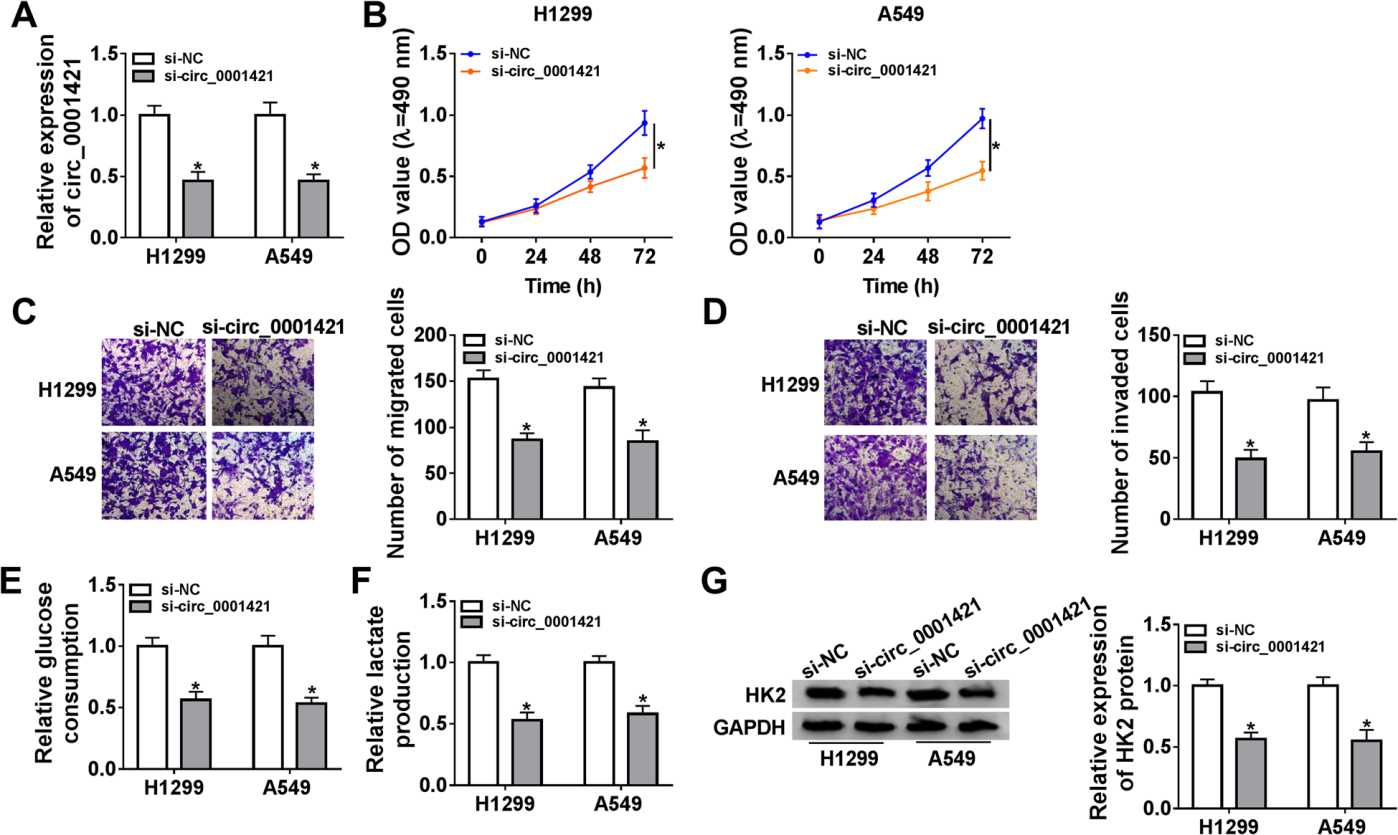
Knockdown of circ_0001421 repressed LC cell proliferation, migration, invasion and glycolysis in vitro. H1299 and A549 cells were transfected with si-NC or si-circ_0001421. **a** Circ_0001421 expression was measured by qRT-PCR. **b** MTT assay was performed to assess cell proliferation. **c-d** Cell migration and invasion were determined by Transwell assay. **e-f** Glucose consumption and lactate production were examined by a Glucose assay kit and a Lactic Acid assay kit. **g** Western blot was performed to measure the protein expression of HK2. *P < 0.05

Subsequently, several experiments were performed to explore the function of circ_0001421 in LC. As shown in Fig. 2a, the lower expression of circ_0001421 was found in A549 and H1299 cells by transfection of si-circ_0001421 relative to that in si-NC-transfected cells. MTT assay demonstrated that circ_0001421 inhibition retarded proliferation in A549 and H1299 cells comparison with the control group (Fig. 2b). Transwell assay results manifested that circ_0001421 knockdown notably curbed cell migration and invasion of A549 and H1299 cells (Fig. 2c-d). Glucose consumption and lactate production assays were performed to assess the effect of circ_0001421 on glycolysis. Circ_0001421 knockdown significantly reduced glucose consumption and lactate production in A549 and H1299 cells (Fig. 2e-f). Meanwhile, HK2 protein expression in si-circ_0001421 transfected A549 and H1299 cells was declined (Fig. 2g). Collectively, these results supported that circ_0001421 promoted in LC cell proliferation, migration, invasion, glycolysis, and functioned as an oncogenic circRNA.

### Circ_0001421 could act as a sponge for miR-4677-3p

**Fig. 3:**
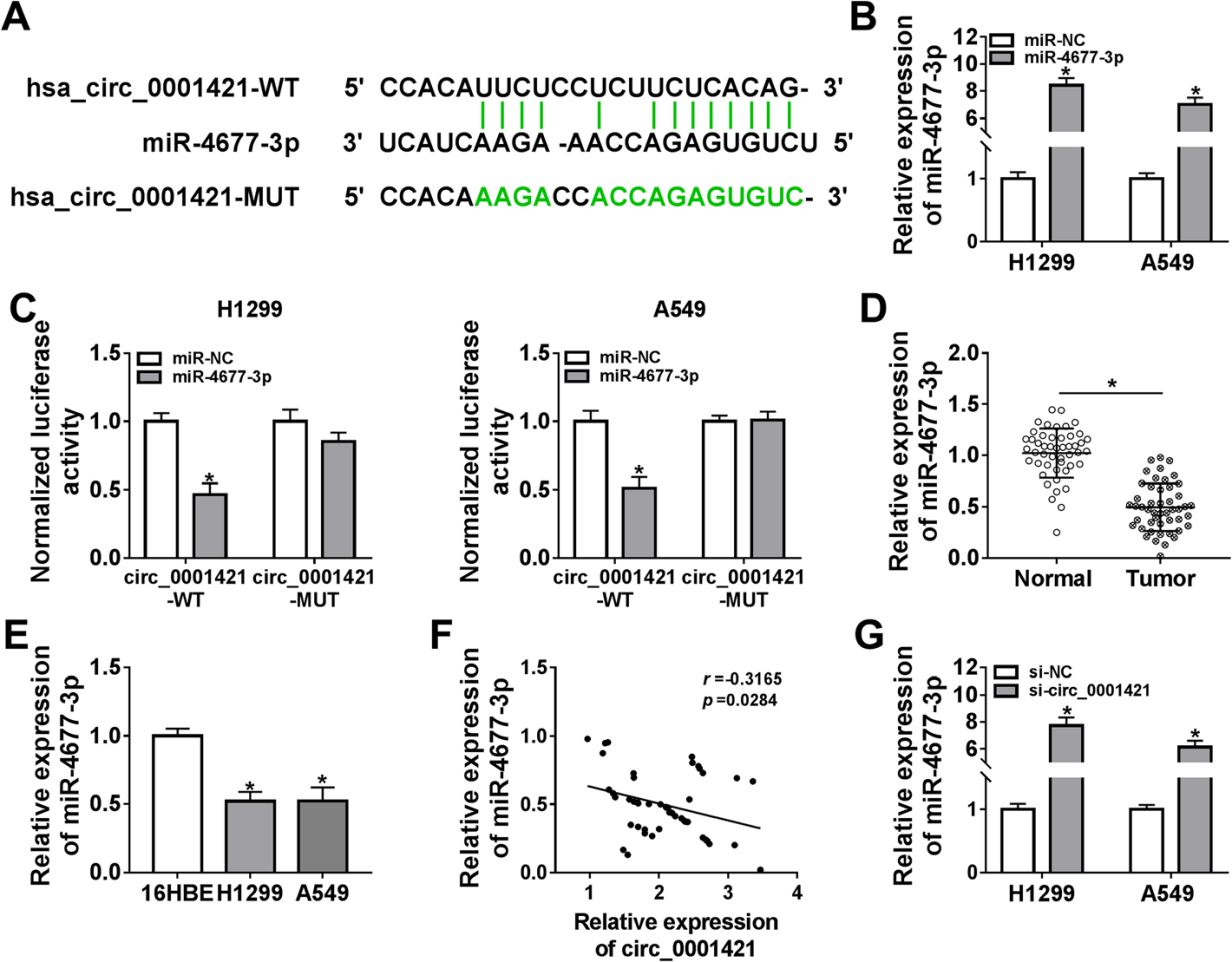
Circ_0001421 could act as a sponge for miR-4677-3p. **a** The predicted binding sites between circ_0001421 and miR-4677-3p were shown. **b** The expression of miR-4677-3p in H1299 and A549 cells transfected with miR-NC or miR-4677-3p was detected by qRT-PCR. **(C)** Dual-luciferase reporter assay was performed to verify the relationship between circ_0001421 and miR-4677-3p in H1299 and A549 cells. **d** miR-4677-3p expression in tumor and adjacent normal tissues was examined by qRT-PCR. **e** qRT-PCR was used to determine miR-4677-3p expression in A549, H1299 and 16HBE cells. **f** The correlation between circ_0001421 and miR-4677-3p expression was analyzed in LC tissues by Pearson coefficient analysis. **g** miR-4677-3p expression in H1299 and A549 cells transfected with si-NC or si-circ_0001421 was measured by qRT-PCR. *P < 0.05

To investigate the molecular mechanism of circ_0001421 regulating the progression of LC cells, Starbase 3.0 was employed to screen the target miRNAs sponged by circ_0001421. The results showed that there were binding sites between circ_0001421 and miR-4677-3p (Fig. 3a), and the relationship between them was then confirmed by dual-luciferase reporter assay. The expression of miR-4677-3p was increased in A549 and H1299 cells by transfection of miR-4677-3p (Fig. 3b). Overexpression of miR-4677-3p drastically degraded the luciferase activity of circ_0001421-WT, but did not distinctly reduce the luciferase activity of circ_0001421-MUT in A549 and H1299 cells (Fig. 3c). Then, we examined miR-4677-3p expression in LC tissues by qRT-PCR. As displayed in Fig. 3d, miR-4677-3p expression was down-regulated in tumor tissues compared to normal tissues. In addition, miR-4677-3p expression was exceptionally decreased in A549 and H1299 cells (Fig. 3e). The correlation between circ_0001421 and miR-4677-3p expression in 48 LC patients was then analyzed and a significant negative correlation between them was observed (Fig. 3f). We also found that knockdown of circ_0001421 up-regulated miR-4677-3p expression in A549 and H1299 cells (Fig. 3g). We concluded that circ_0001421 could target miR-4677-3p to negatively regulate miR-4677-3p expression in LC cells.

### Circ_0001421 regulated LC cell progression by targeting miR-4677-3p in LC cells

**Fig. 4:**
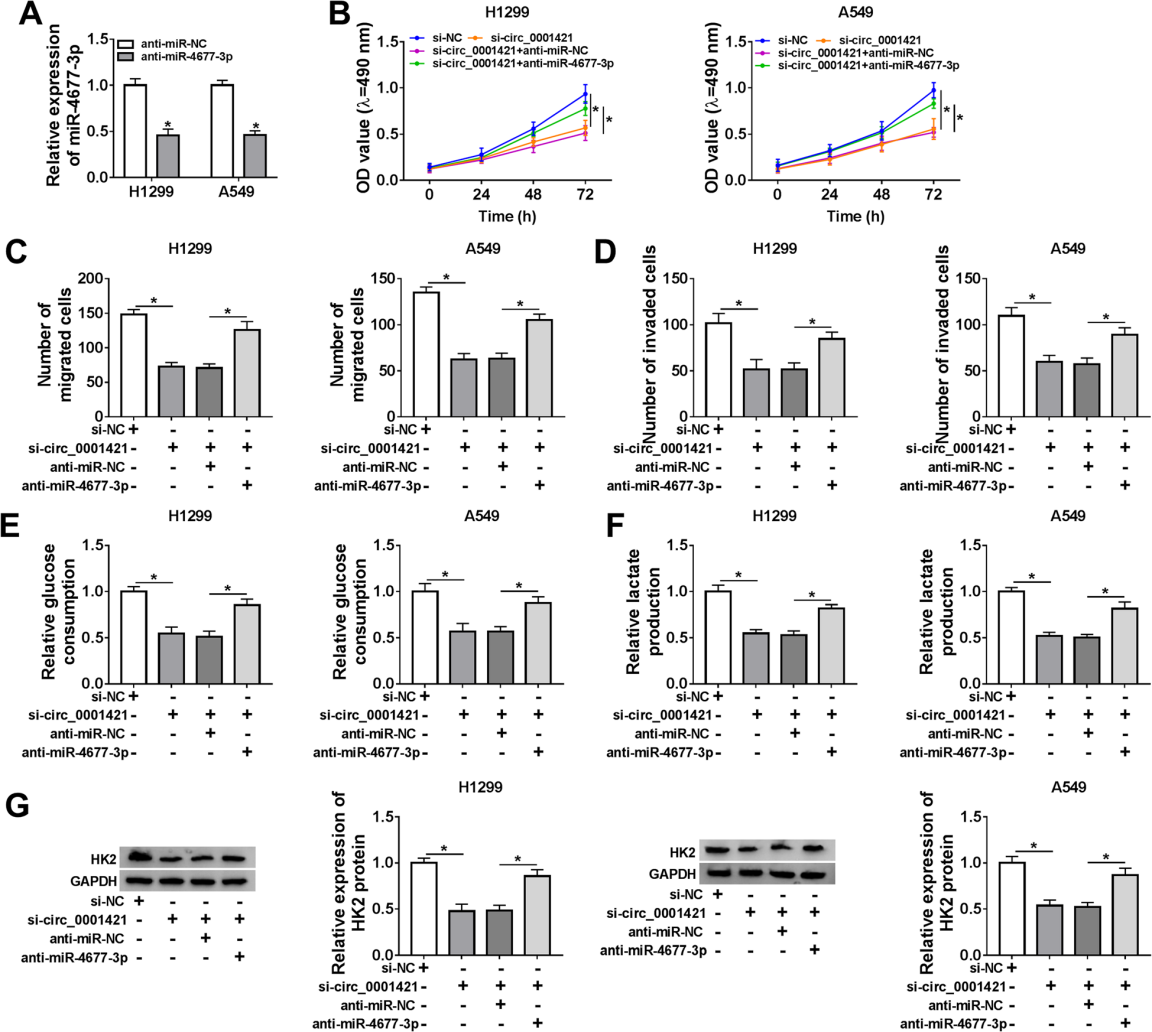
Circ_0001421 regulated LC cell progression by adsorbing miR-4677-3p in LC cells. **a** The expression of miR-4677-3p in H1299 and A549 cells transfected with anti-miR-NC or anti-miR-4677-3p was detected by qRT-PCR. **b-g** H1299 and A549 cells were transfected with si-NC, si-circ_0001421, si-circ_0001421 + anti-miR-NC or si-circ_0001421 + anti-miR-4677-3p. **b** MTT assay was employed to analyze cell proliferation. **c-d** Cell migration and invasion were evaluated by Transwell assay. **e-f** Glucose consumption and lactate production were assessed by a Glucose assay kit and a Lactic Acid assay kit. **g** Western blot was used to check the protein expression of HK2. *P < 0.05

To understand whether miR-4677-3p was involved in circ_0001421 regulation of cell biological behaviors, si-circ_0001421 and anti-miR-4677-3p were co-transfected into A549 and H1299 cells. The results showed that miR-4677-3p was inhibited by transfection of anti-miR-4677-3p in A549 and H1299 cells than cells transfected anti-miR-NC (Fig. 4a). MTT and Transwell assay indicated that miR-4677-3p inhibition reversed the inhibition effects of si-circ_0001421 on the proliferation (Fig. 4b), migration (Fig. 4c) and invasion (Fig. 4d) of A549 and H1299 cells. Moreover, glucose consumption and lactate production were decreased by circ_0001421 knockdown and then recovered by miR-4677-3p inhibition knockdown in A549 and H1299cells (Fig. 4e-f), and the inhibition effect of si-circ_0001421 on HK2 expression could be reversed by silencing miR-4677-3p (Fig. 4g). These findings suggested that circ_0001421 executed its carcinogenic role in the progression of LC cells by targeting miR-4677-3p.

### CDCA3 was a target gene of miR-4677-3p in LC

**Fig. 5:**
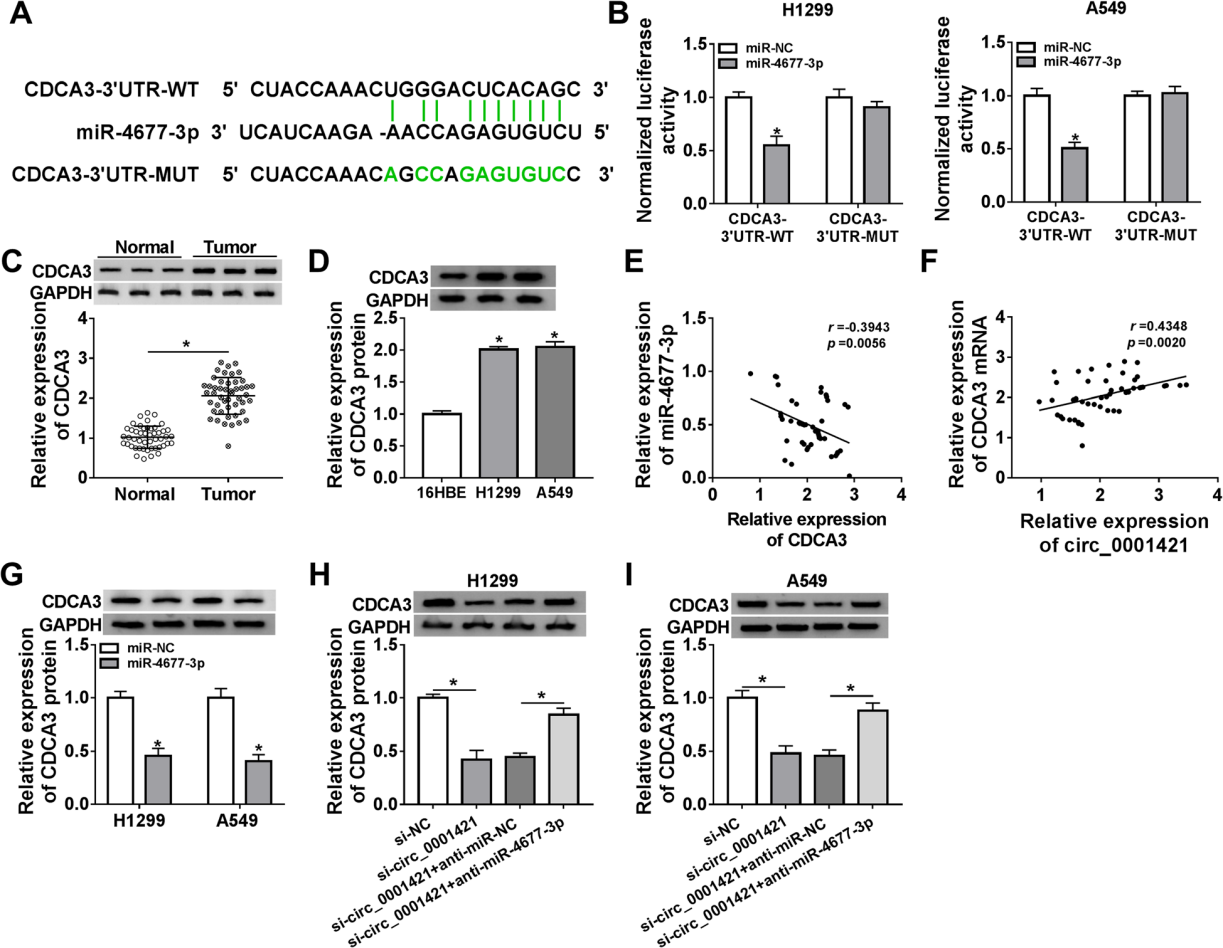
CDCA3 was a target gene of miR-4677-3p in LC. **a** The predicted binding sites between miR-4677-3p and CDCA3 were shown. **b** The relationship between miR-4677-3p and CDCA3 in H1299 and A549 cells was analyzed by dual-luciferase reporter assay. **c-d** The protein of CDCA3 in LC tissues and cells versus that in normal tissues and cells was examined by western blot. **e** The correlation between miR-4677-3p and CDCA3 expression was appraised in LC tissues through Pearson coefficient analysis. **f** The correlation between miR-4677-3p and circ_0001421 level was appraised in LC tissues through Pearson coefficient analysis. **g** The protein of CDCA3 in H1299 and A549 cells transfected with miR-4677-3p or miR-NC was measured by western blot. **h-i** The protein of CDCA3 in H1299 and A549 cells transfected with si-NC, si-circ_0001421, si-circ_0001421 + anti-miR-NC or si-circ_0001421 + anti-miR-4677-3p was determined by western blot. *P < 0.05

MiRNAs often play biological roles by binding to the 3’UTR of mRNAs [20]. To verify the target mRNAs of miR-4677-3p, Starbase 3.0 was employed. As shown in Fig. 5a, the 3’UTR of CDCA3 contained miR-4677-3p binding sites. To further confirm this prediction, dual-luciferase reporter assay was performed in A549 and H1299 cells. We observed that the luciferase activity of CDCA3–3’UTR-WT was reduced in A549 and H1299 cells transfected with miR-4677-3p, while the luciferase activity of CDCA3–3’UTR-MUT was no obvious variation (Fig. 5b). To explore the role of CDCA3 in LC, CDCA3 expression in LC tissues and cells was detected by western blot. As shown in Fig. 5c-d, CDCA3 protein expression in LC tissues and cells was drastically increased compared to normal tissues and cells. Unsurprisingly, CDCA3 expression was negatively correlated with miR-4677-3p expression and positively related to circ_0001421 level (Fig. 5e-f). Besides, miR-4677-3p overexpression reduced the protein expression of CDCA3 in A549 and H1299 cells (Fig. 5g). Western blot results also revealed that circ_0001421 knockdown degraded CDCA3 expression, while this effect could be weakened by miR-4677-3p deficiency in A549 and H1299 cells (Fig. 5h-i). Collectively, miR-4677-3p could bind to CDCA3 and regulate its expression in LC.

### CDCA3 overexpression reversed the inhibitory effects of circ_0001421 knockdown on LC cell proliferation, migration, invasion and glycolysis

**Fig. 6:**
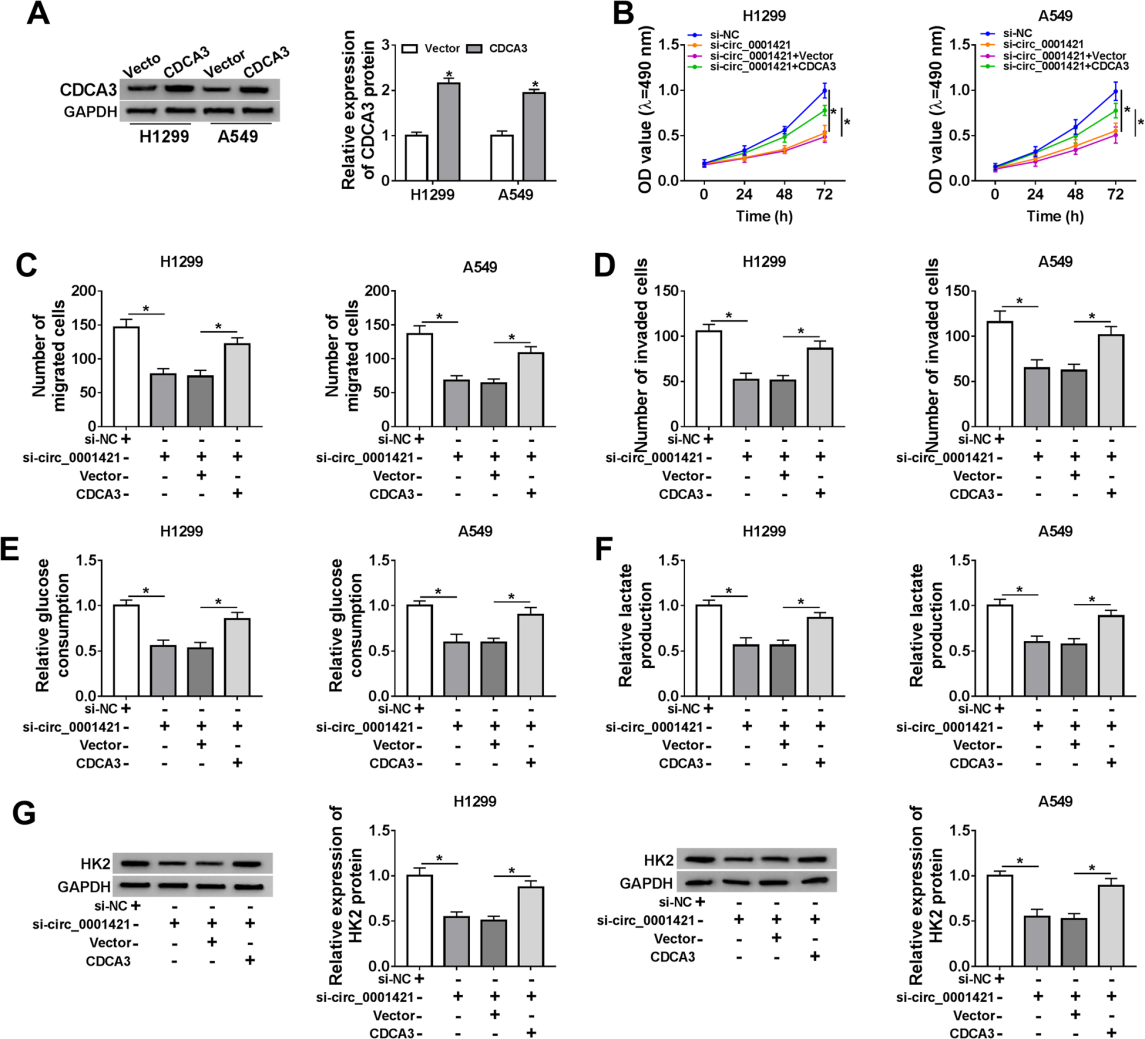
CDCA3 overexpression reversed the inhibitory effects of circ_0001421 knockdown on LC cell proliferation, migration, invasion and glycolysis. **a** The protein of CDCA3 in H1299 and A549 cells transfected with Vector or CDCA3 was detected by western blot. **b-g** H1299 and A549 cells were transfected with si-NC, si-circ_0001421, si-circ_0001421 + Vector or si-circ_0001421 + CDCA3. **b** Cell proliferation was assessed by MTT assay. **c-d** Cell migration and invasion were assessed by Transwell assay. **e-f** Glucose consumption and lactate production were appraised via a Glucose assay kit and a Lactic Acid assay kit. **g** Western blot was performed to check the protein expression of HK2. *P < 0.05

Considering that circ_0001421 could regulate the expression of CDCA3, we then investigated whether circ_0001421 played a role in LC cells by regulating CDCA3. As shown in Fig. 6a, CDCA3 protein expression in A549 and H1299 cells transfected with CDCA3 was significantly increased than the control group. In above data, we demonstrated that circ_0001421 knockdown impeded cell proliferation (Fig. 6b), migration (Fig. 6c) and invasion (Fig. 6d), all of which could be neutralized by overexpression of CDCA3 in A549 and H1299 cells. Meanwhile, CDCA3 overexpression offset the repression of circ_0001421 knockdown on glucose consumption (Fig. 6e), lactate production (Fig. 6f) and the protein of HK2 (Fig. 6g) in A549 and H1299 cells. These results suggested that circ_0001421 could regulate the development of LC cells by regulating CDCA3.

### Knockdown of circ_0001421 inhibited cell growth of LC cells in vivo

**Fig. 7:**
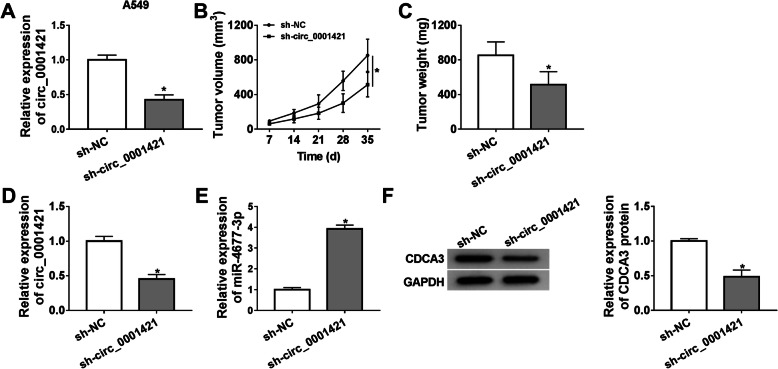
Knockdown of circ_0001421 inhibited cell growth of LC cells in vivo. **a** The expression of circ_0001421 was detected by qRT-PCR in A549 cells. **b-c** The tumor volume and weight in the groups of sh-NC and sh-circ_0001421 were shown. **d-e** The expression levels of circ_0001421 and miR-4677-3p in that tumor were measured by qRT-PCR. **f** The protein of CDCA3 was examined by western blot. *P < 0.05

To further certify the promotion of circ_0001421 in LC development in vivo, the xenograft experiments were carried out in nude mice. Our data showed that circ_0001421 was downregulated in A549 cells (Fig. 7a). As demonstrated in Fig. 7b-c, the volume and weight of the tumors in sh-circ_0001421 group were smaller than those tumors in sh-NC group. Meanwhile, qRT-PCR indicated that the expression of circ_0001421 was reduced, while miR-4677-3p expression was increased in tumors of sh-circ_0001421 group (Fig. 7d-e). The protein expression of CDCA3 in sh-circ_0001421 group was markedly reduced (Fig. 7f). In summary, these results identified that inhibition of circ_0001421 restrained LC cell growth in vivo.

## Discussion

Despite major breakthroughs in the treatment for LC in recent years, LC still poses a great threat to human health. Many reports demonstrated that abnormal circRNA expression was related to the development of a variety of human cancers, including LC [21, 22]. A previous study claimed that circ_0001421 was up-regulated in NSCLC tissues [12]. In this research, we manifested the high expression of circ_0001421 in LC tissues and cell lines. Furthermore, we explored the functional effects of circ_0001421 and revealed that circ_0001421 deficiency restrained cell proliferation, migration and invasion, and reduced glucose consumption, lactate production and the protein of HK2, suggesting silencing circ_0001421 also inhibited cell glycolysis. The lactate produced during glycolysis could to disrupt the extracellular matrix, leading to the rapid cell proliferation, inhibiting apoptosis, and facilitating cell metastasis [23]. These results elucidated that circ_0001421 might play a role in promoting the development of LC.

Mechanistically, it has been determined that circRNAs functioned as competing endogenous RNAs (ceRNAs) of miRNAs to regulate mRNA expression [24]. For instance, circRNA-002178 served as a ceRNA of miR-34 to promote PDL1 expression in lung adenocarcinoma [25]. Circ-ZKSCAN1 modulated FAM83A expression via sponging miR-330-5p in NSCLC cells [26]. In this paper, miR-4677-3p was a downstream target of circ_0001421. Furthermore, miR-4677-3p expression and role have been mentioned in the study of Han et al., lncRNA LINC02418 elevated proliferation and invasion of NSCLC cells via sponging miR-4677-3p and regulating SEC61G [27]. As expected, circ_0001421 could negatively modulate miR-4677-3p expression, while interference with miR-4677-3p abrogated the suppressive effects of circ_0001421 knockdown on cell progression, suggesting that miR-4677-3p has an inhibition effect on the development of LC. Taken together, our results confirmed that circ_0001421 contributed to the development of LC by acting as a miR-4677-3p sponge.

The downstream gene of the circ_0001421/miR-4677-3p axis was further explored. CDCA3 was identified as the target of miR-4677-3p by Starbase 3.0 prediction and dual-luciferase reporter assay. A previous report using a microarray data set from 163 tumor types for gene network analysis, demonstrated that CDCA3 was prominently up-regulated in breast, lung, and ovarian cancers relative to that normal tissues [28]. In line with these results, we revealed that CDCA3 was enhanced in LC tissues and cells. Additionally, we observed that CDCA3 overexpression inverted the inhibition role of circ_0001421 knockdown in LC cell progression. Furthermore, Adams et al. revealed that high expression of CDCA3 was associated with poor prognosis in patients with NSCLC, and interference with CDCA3 inhibited cell proliferation [29]. Consistent with the results in vitro, circ_0001421 suppression impaired the growth of tumor cells in vivo. Simultaneously, we observed that miR-4677-3p expression was increased, while CDCA3 expression was reduced in the stripped tumor tissues of circ_0001421 decreased group.

## Conclusion

The present study revealed that circ_0001421 participated in cell proliferation, migration, invasion and glycolysis in LC cells via modulating the miR-4677-3p/CDCA3 axis. The results of this study might provide the meaningful inspiration for exploring LC progression.

## Data Availability

The data sets used and/or analyzed during the current study are available from the corresponding author on reasonable request.

## Abbreviations

LC: Lung cancer
circRNAs: Circular RNAs
miR-4677-3p: microRNA-4677-3p
CDCA3: Cell division cycle associated 3
qRT-PCR: quantitative real-time polymerase chain reaction
MTT: Methyl thiazolyl tetrazolium
HK2: Hexokinase 2
ncRNA: non-coding RNA
miRNAs: microRNAs
FBS: Fetal bovine serum
cDNA: complementary DNA
SDS-PAGE: Sodium dodecyl sulfate polyacrylamide gel electrophoresis
